# Comparison of the therapeutic effects of three internal fixation methods for transverse fractures of the patella

**DOI:** 10.3389/fsurg.2025.1633670

**Published:** 2025-07-30

**Authors:** Xiao-Dong Li, Kewei Du

**Affiliations:** Department of Orthopedics, Shidong Hospital Affiliated to University of Shanghai for Science and Technology, Shanghai, China

**Keywords:** tension band with ring-pins, cannulated screws, transverse fractures of the patella, internal fixation, fractures abstract, surgery

## Abstract

**Background:**

This study aimed to evaluate the treatment effects of three internal fixation methods, including tension band with K-wires, tension band with cannulated screws and tension band with ring-pins on transverse fractures of the patella.

**Methods:**

Patellar fracture patients treated in Shanghai Yangpu District Shidong Hospital from March 2016 to January 2023 were divided into three groups based on the internal fixation method: the ring-pins group (tension band with ring-pins), the K-wires group (tension band with K-wires), and the cannulated screws group (tension band with cannulated screws). Surgery duration, hemoglobin decrease, excellent rate of knee joint function evaluation at postoperative 3 months and 12 months, VAS scores before surgery and at 3 and 12 months after surgery, postoperative complication rate, and removal rate of internal fixation were compared among the three groups of patients.

**Results:**

No significant differences were observed in operation time and the degree of hemoglobin decrease before and after operation among the three groups (*P* > 0.05). There was a statistical difference in the excellent and good rate of Böstman's score among the three groups of patients 3 months after surgery (*P* < 0.05). The excellent and good rates of knee joint scores in the ring-pins group and the cannulated screws group in 3 months after surgery were higher than in the K-wires group, and no significant differences were observed in the excellent and good rates of Böstman's score among the three groups at 12 months after surgery (*P* > 0.05). No significant differences were observed in VAS scores across groups before and after surgery (*P* > 0.05). The VAS scores at 3 months and 12 months after surgery were lower than those before surgery (*P* < 0.001). Significant differences were observed in the incidence of postoperative complications (*P* < 0.05) and hardware removal rates (*P* < 0.001) among the three groups. The ring-pins group and the cannulated screws group had fewer postoperative complications than the K-wires group.

**Conclusion:**

Compared with K-wires and cannulated screws, ring-pins have the advantages of wide application range, low postoperative complication rate, and easy removal of implants. Treatment experience, low surgical complexity, and good postoperative knee joint function recovery have obvious clinical application value.

## 1 Introduction

The patella is an essential component of normal knee function, and fractures of the patella account for 1%–1.7% of all fractures in adults (1). These fractures are most common in patients aged 20–50 years and are often caused by falls or traffic accidents. Transverse fractures are the most common type, accounting for approximately 50% of patellar fractures, and can usually be identified through imaging. Surgical intervention is indicated when the displacement is ≥3 mm, the articular surface defect is >2 mm, or there is dysfunction in knee extension (2). Currently, tension band fixation is a commonly used surgical approach (3), with the tension band with K-wires and tension band with cannulated screws being widely used and considered classic methods. However, both techniques have notable drawbacks, such as the tendency for K-wires to back out or for cannulated screws cause iatrogenic bone damage and increased surgical complexity during removal. With advancements in materials, ring-pins combining the advantages of both classic methods while mitigating some of their shortcomings (4). This technique has been gradually adopted in clinical practice in recent years. Lee et al. demonstrated that all three fixation techniques provide adequate biomechanical stability for normal daily activities (5), Kim et al. reported that the modified technique provides dual benefits: enhanced implant stability and reduced propensity for causing soft tissue irritation (6).

This study retrospectively analyzed 101 cases of transverse patellar fractures treated at our hospital. We compared the therapeutic outcomes of three tension band fixation methods, including surgical duration, hemoglobin level decrease, knee function (Böstman scores), pain relief (VAS scores), complication rates, and implant removal rates. Our findings provide evidence-based recommendations for optimizing fixation strategies.

## 2 Materials and methods

### 2.1 Subjects of the study

Patellar fracture patients admitted to Shanghai Yangpu District Shidong Hospital from March 2016 to January 2023 were included in the present retrospective study, comparing the clinical efficacy of three different tension band internal fixation methods. Inclusion criteria were: (1) fresh transverse patellar fractures caused by trauma; (2) aged ≥18 years, without major underlying medical conditions or mental illness, and with regular postoperative follow-up. (3) Patients who were self-sufficient and had normal knee function before the fracture. Exclusion criteria were: (1) patients with major underlying medical conditions or mental illness; (2) patients with concurrent fractures in other parts of the body; (3) patients with preoperative skin abrasions or infections at the surgical site; (4) patients lost to follow-up after surgery. This study was approved by the Ethics Committee of Shanghai Yangpu District Shidong Hospital (2023-061-01).

A total of 101 patients with transverse patellar fractures treated surgically in our hospital from March 2016 to January 2023 were included in the study. The cohort consisted of 39 males and 62 females, aged 25–90 years (average age: 64.1 years). Based on the internal fixation method, patients were divided into three groups: the ring-pins group (tension band with ring-pins, 19 cases), the K-wires group (tension band with K-wires, 51 cases), and the cannulated screws group (tension band with cannulated screws, 31 cases). There were no significant differences in sex, age, or time from injury to surgery among the three groups (*P* > 0.05). All fractures were located in the middle or lower third of the patella, ensuring group comparability (Table 1).

**Table 1:** Baseline characteristics of the three groups.

Group	Cases	Sex, male/female	Age, years	Time from injury to surgery, days
The ring-pins group	19	7/12	63.0 ± 15.5	3.7 ± 2.7
The K-wires group	51	19/32	65.4 ± 13.8	3.4 ± 3.0
The cannulated screws group	31	13/18	62.6 ± 9.6	3.1 ± 2.1
*χ*^2^/F value		0.207	0.522	0.742
*P*		0.902	0.595	0.690

### 2.2 Surgical methods

All patients underwent the same preoperative preparation, including elevation and immobilization of the affected knee, brace fixation, and routine preoperative examinations. Patients received either spinal or general anesthesia and were placed in the supine position. The surgical area was sterilized and draped, and the procedure was performed under C-arm fluoroscopy.

#### 2.2.1 Preoperative examination

All patients underwent knee x-rays (anteroposterior and lateral views) and CT scans to assess the fracture type and displacement. A comprehensive evaluation of the patient's overall condition and pre-injury knee function was also conducted.

#### 2.2.2 Surgical procedure

A longitudinal incision was made over the patella, and the fracture site and intra-articular hematoma were cleared. The fracture ends were reduced and temporarily fixed with reduction forceps. The ring-pins group: Two φ2.0 mm ring-pins were inserted from the inferior to the superior pole of the patella. A cable was passed through the pinhole in a figure-of-eight configuration. The knee was flexed and extended to ensure stable fixation, and fluoroscopy confirmed satisfactory reduction and proper positioning of the ring-pins and cable. The ends of the ring-pins were bent and cut. The K-wires group: Two φ2.0 mm K-wires were inserted from the inferior to the superior pole of the patella. A cable was used in a figure-of-eight configuration. The knee was flexed and extended to ensure stable fixation, and fluoroscopy confirmed satisfactory reduction and proper positioning of the K-wires and cable. The ends of the K-wires were bent and cut. The cannulated screws group: Two guide pins were inserted from the inferior to the superior pole of the patella. Two φ3.5 mm cannulated screws were placed over the guide pins, and a cable was used in a figure-of-eight configuration. The knee was flexed and extended to ensure stable fixation, and fluoroscopy confirmed satisfactory reduction and proper positioning of the screws and cable.

#### 2.2.3 Postoperative management

Prophylactic antibiotics were administered within 1 h before surgery and discontinued within 24 h after the procedure. Routine pain management and anti-swelling treatments were provided. Blood biochemical tests were repeated, and functional exercises were initiated on the first postoperative day, with knee flexion ≥90° and full extension. Gradual weight-bearing and knee flexion-extension exercises were performed at 6–8 weeks postoperatively.

### 2.3 Evaluation criteria

All patients were followed up for 3–12 months postoperatively. Radiologic exams were conducted to assess fracture healing and knee function. The primary outcomes included: (1) Intraoperative conditions: surgery duration (from skin incision to wound closure), blood loss (hemoglobin decrease before and after surgery), and hospital stay. (2) Excellent and good rates of knee function Böstman scores at 3 and 12 months postoperatively (Böstman score (7): assessed based on knee range of motion (6 points), pain level (6 points), impact on daily activities (4 points), muscle atrophy (2 points), need for crutches (2 points), joint effusion (2 points), weakness during walking (2 points), and stair-climbing ability (2 points). A score of 20 is poor, 20–27 is good, and 28–30 is excellent. (3) Preoperative and postoperative Visual Analog Scale(VAS) scores at 3 and 12 months (8): participants mark their pain level on a continuous line, with 0 indicating no pain, 1–3 mild pain, 4–6 moderate pain, 7–9 severe pain, and 10 unbearable pain). (4) Postoperative complication rates (e.g., wire migration, soft-tissue irritation) and hardware removal rates.

### 2.4 Statistical analysis

Statistical analysis was performed using jamovi 2.3.28.0 software. Categorical data were expressed as percentages and analyzed using the *χ*^2^ test. Continuous data were expressed as mean ± standard deviation. The Shapiro–Wilk test was used to assess normality. Data conforming to a normal distribution were analyzed using the *F*-test, while non-normally distributed data were analyzed using the Kruskal–Wallis *H*-test. Intra-group comparisons were performed using repeated measures ANOVA. *P* < 0.05 was considered statistically significant.

## 3 Results

### 3.1 Surgical and postoperative recovery conditions

All patients were followed up for 12–18 months (mean: 15 months). No infections, non-unions, or internal fixation failures occurred. All wounds healed well, with no iatrogenic injuries. The average hospital stay was 10.9 days. There were no significant differences in surgery duration, hemoglobin decrease, or hospital stay among the three groups (*P* > 0.05) (Table 2).

**Table 2:** Comparison of intraoperative and postoperative conditions among three groups.

Group	Surgery duration, minutes	Hemoglobin decrease, g/L	Hospital stay, days
The ring-pins group	89.1 ± 40.8	1.8 ± 8.4	10.0 ± 2.2
The K-wires group	82.8 ± 35.0	5.8 ± 11.4	11.9 ± 3.4
The cannulated screws group	71.5 ± 23.7	7.5 ± 9.5	9.8 ± 2.7
*χ*^2^/F value	1.577	1.649	9.001
*P*	0.454	0.199	0.011

### 3.2 Postoperative knee function Böstman scores

There was a significant difference in the excellent and good rates of Böstman scores among the three groups at 3 months postoperatively (*P* < 0.05). However, there was no significant difference at 12 months postoperatively (*P* > 0.05) (Table 3). In the K-wires group, 4 out of 8 patients with poor functional scores at 3 months showed improvement to good after hardware removal at 12 months.

**Table 3:** Proportion of excellent/good outcomes of postoperative knee function Böstman scores among three groups.

Group	3 months before hardware removal	12 months before hardware removal
The ring-pins group	100%	100%
The K-wires group	84%	92%
The cannulated screws group	100%	100%
*χ* ^2^	8.518	4.083
*P*	0.014	0.130

### 3.3 Postoperative pain conditions

There were no significant differences in VAS scores before and after surgery among the three groups (*P* > 0.05). However, the VAS scores at 3 and 12 months postoperatively were significantly lower than preoperative scores (*P* < 0.001) (Table 4).

**Table 4:** Preoperative and postoperative VAS scores among three groups.

Group	Preoperative	3 months post-op	12 months post-op	*F* value	*P*
The ring-pins group	5.11 ± 1.15	1.63 ± 0.50	1.05 ± 0.23	175.054	<0.001
The K-wires group	4.88 ± 0.99	1.65 ± 0.59	1.12 ± 0.33	477.586	<0.001
The cannulated screws group	4.87 ± 0.76	1.68 ± 0.54	1.03 ± 0.18	405.583	<0.001
*χ* ^2^	0.831	0.117	2.133		
*P*	0.660	0.943	0.344		

### 3.4 Postoperative complications

There were significant differences in postoperative complication rates (*P* < 0.05) and hardware removal rates (*P* < 0.001) among the three groups (Table 5). In the ring-pins group, there was 1 case of soft-tissue irritation. In the K-wires group, there were 6 cases of soft-tissue irritation and 6 cases of wire migration. In the cannulated screws group, there were 2 cases of soft-tissue irritation. The hardware removal rate was correlated with postoperative complications (*P* < 0.001). Considering the influence of age, we compared the incidence of postoperative complications between those >70 and **≤**70 years old, both within and across groups, revealed no statistically significant differences (*P* > 0.05) (Table 6).

**Table 5:** Comparison of postoperative complications and hardware removal rates among three groups.

Group	Postoperative complications	Implant removal, %
The ring-pins group	5.3% ^a^/0%^b^	21.1%^c^
The K-wires group	11.8% ^a^/11.8%^b^	74.5%^c^
The cannulated screws group	6.5%^a^/0%^b^	22.6%^c^
*χ*^2^ value	6.148	27.881
*P*	0.046	<0.001

^a^ Soft-tissue irritation.

^b^ Wire migration.

^c^ Proportion of all patients in each group.

**Table 6:** Comparison of postoperative complications among the three groups with age stratification (>70 years).

Group	Cases	Postoperative complications	*χ*^2^ value	*P*
The ring-pins group	5	0% ^a^/0%^b^	0.377^c^	0.539^c^
The K-wires group	11	12.5%^a^/18.8%^b^	0.772^c^	0.379^c^
The cannulated screws group	5	0% ^a^/0%^b^	0.411^c^	0.521^c^
*χ*^2^ value		3.869		
*P*		0.144		

^a^ Soft-tissue irritation.

^b^ Wire migration.

^c^ Comparing >70 years cases vs. ≤70 years cases.

**Figure 1:**
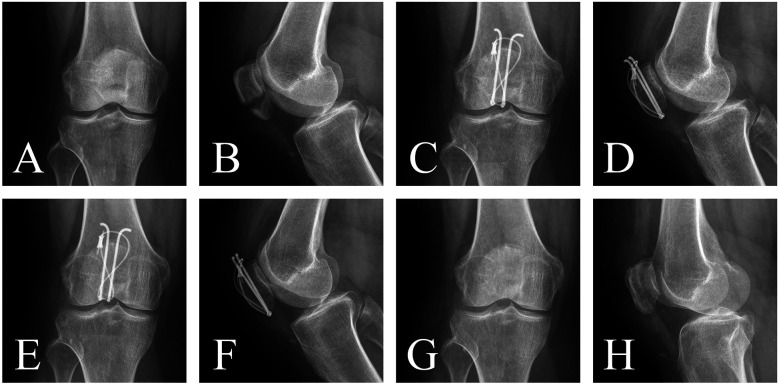
The ring-pins group: **(A**,**B)** preoperative anteroposterior and lateral x-rays showing significant fracture displacement. **(C**,**D)** Postoperative 3-month anteroposterior and lateral x-rays demonstrating satisfactory reduction, smooth articular surface, and no wire migration. **(E**,**F)** Postoperative 15-month anteroposterior and lateral x-rays showing blurred fracture lines. **(G**,**H)** Postoperative 15-month anteroposterior and lateral x-rays after hardware removal, showing complete fracture healing.

Case 1: A 50-year-old female patient presented with right knee pain and limited mobility for 1 day following a fall. She was admitted with a diagnosis of right patellar fracture. Under general anesthesia with intubation, she underwent open reduction and internal fixation of the patellar fracture using a tension band with ring-pins band. Postoperatively, there were no signs of infection, wire migration, or breakage. After 15 months of follow-up, the hardware was removed, and the patient showed good fracture healing (Figure 1).

**Figure 2:**
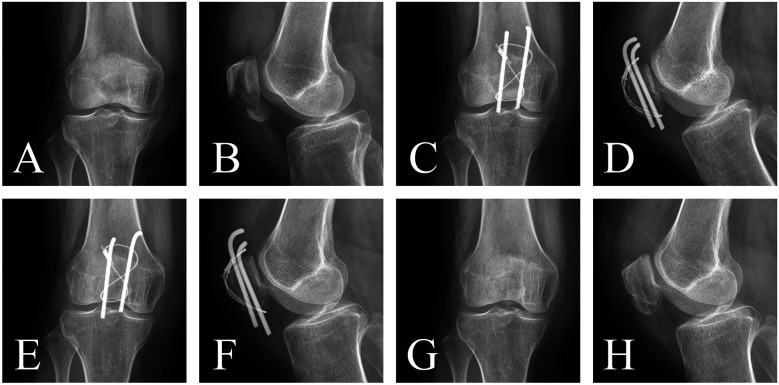
The K-wires group: **(A**,**B)** preoperative anteroposterior and lateral x-rays showing fracture displacement and angulation. **(C**,**D)** Postoperative 3-month anteroposterior and lateral x-rays demonstrating satisfactory reduction, correction of angulation, and no wire migration. **(E**,**F)** Postoperative 11-month anteroposterior and lateral x-rays showing blurred fracture lines. **(G**,**H)** Postoperative 12-month anteroposterior and lateral x-rays after hardware removal, showing complete fracture healing.

Case 2: A 62-year-old female patient presented with right knee pain and limited mobility for 5 days following a fall. She was admitted with a diagnosis of right patellar fracture. Under general anesthesia with intubation, she underwent open reduction and internal fixation of the patellar fracture using an Tension band with K-wires. Postoperatively, there were no signs of infection, wire migration, or breakage. After 12 months of follow-up, the hardware was removed, and the patient showed good fracture healing (Figure 2).

**Figure 3:**
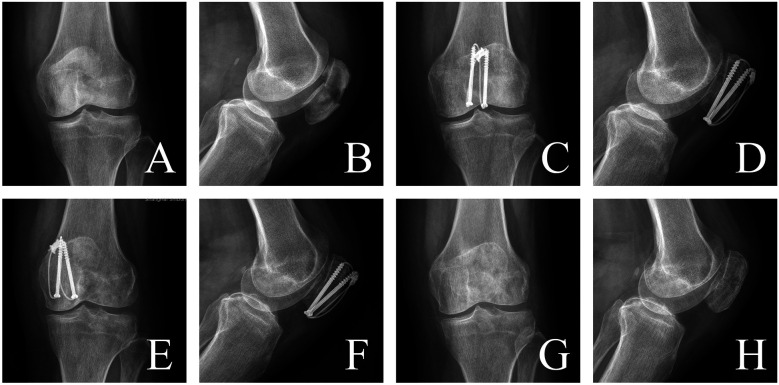
The cannulated screws group: **(A**,**B)** preoperative anteroposterior and lateral x-rays showing fracture angulation. **(C**,**D)** Postoperative 3-month anteroposterior and lateral x-rays demonstrating correction of angulation, smooth articular surface, and no screw migration. **(E**,**F)** Postoperative 12-month anteroposterior and lateral x-rays showing blurred fracture lines. **(G**,**H)** Postoperative 13-month anteroposterior and lateral x-rays after hardware removal, showing complete fracture healing.

Case 3: A 59-year-old female patient presented with left knee pain and limited mobility for 1 day following a fall. She was admitted with a diagnosis of left patellar fracture. Under general anesthesia with intubation, she underwent open reduction and internal fixation of the patellar fracture using a tension band with cannulated screws. Postoperatively, there were no signs of infection, screw migration, or breakage. After 13 months of follow-up, the hardware was removed, and the patient showed good fracture healing (Figure 3).

## 4 Discussion

Patellar fractures are common traumatic fractures in clinical practice, requiring rigid fixation and anatomical reduction to achieve early functional exercise (9), prevent knee stiffness, and restore knee extension function (10). Currently, different internal fixation methods and surgical approaches are used for different types of patellar fractures. Tension band internal fixation is the standard surgical method for patellar fractures (11), with the ring-pins, K-wires and cannulated screws being commonly used. However, there is no widespread consensus on which internal fixation method is superior. The ring-pins group, as a newer technique, combines the advantages of the tension band with K-wires and the tension band with cannulated screws and has been gradually adopted in clinical practice. This study compared the three methods in terms of several dimensions based on 101 cases of transverse patellar fractures treated surgically in our hospital, providing a reference for the selection of surgical methods in clinical practice.

This study found no significant differences in fracture healing time among the three internal fixation methods. The tension band with cannulated screws had no risk of wire slippage, as the screw and wire form a single unit that adheres closely to the bone, providing strong resistance to wire migration (12). This method is less likely to result in fixation failure or fracture displacement and is simple to perform. It is suitable for patellar fractures with large fragments and minimal comminution. However, the drilling process can cause bone damage (13); Bone ingrowth into screws may complicate removal, and cable laceration by screw threads may lead to retained hardware. The tension band with K-wires is simple to perform, cost-effective, and causes minimal bone damage, making it suitable for comminuted fractures. However, K-wires are prone to migration and loosening during and after surgery, leading to unstable fixation (14–17). As surgeons may bend both K-wires ends for stability, removal necessitates cutting a bent end through extended incisions. In this study, 6 cases of wire migration were observed. Additionally, the long ends of the K-wires can irritate the surrounding soft-tissues (18), as seen in 6 cases in this study. However, in our study, 74.5% of patients in the K-wires group underwent hardware removal after fracture healing, which imposed additional economic burdens and surgical risks on these patients. The ring-pins group is similar in surgical technique to the tension band with K-wires, but the wire passing through the ring-pins forms a single unit, which reduced the risk of migration (6, 19) and fixation failure (4, 19). The shorter ends of the K-wires cause less soft-tissue irritation, effectively alleviating postoperative knee pain and allowing for early functional rehabilitation (20). This method combines the simplicity and minimal bone damage of the tension band with K-wires with the advantages of the tension band with cannulated screws.

In this study, there were no significant differences in surgery duration, hemoglobin decrease, time from injury to surgery, or hospital stay among the three surgical methods, and the costs of surgery were similar. This indicates that the complexity of the three surgical techniques is comparable, and the financial burden on patients is similar. The ring-pins group and the K-wires group had slightly longer surgery duration than the cannulated screws group, which may be related to the additional steps of adjusting the depth of the K-wires and bending and cutting their ends. The larger diameter of the cannulated screws causes more bone damage, resulting in a greater hemoglobin decrease in the cannulated screws group compared to the other two groups. Surgeons should consider the patient's bone quality, preoperative hemoglobin levels, and overall condition when selecting the appropriate internal fixation method. The VAS scores before and after surgery showed that all three surgical methods significantly alleviated pain, achieving the surgical goals. The K-wires group had worse knee function scores at 3 months postoperatively compared to the ring-pins group and the cannulated screws group (*P* < 0.05). However, there was no significant difference in knee function scores at 12 months postoperatively, which may be related to the higher complication rates (e.g., wire migration, soft-tissue irritation) and higher hardware removal rates in the K-wires group. In the K-wires group, hardware removal at 12 months significantly improved knee function scores (*P* < 0.001), suggesting that timely hardware removal can alleviate pain and dysfunction caused by complications. The high rate of wire migration in the K-wires group may be related to the following factors: (1) The majority of patients in this study were elderly (mean age: 64.1 years), with a high prevalence of osteoporosis and poor bone quality. (2) The smooth surface of the K-wires and the bending of only one end can lead to loosening after fracture healing. (3) Repeated knee flexion and extension can cause deformation of the K-wires, leading to bone resorption due to pressure on the surrounding bone. For elderly patients with osteoporosis, the ring-pins group and the tension band with cannulated screws can reduce the risk of wire migration. Postoperative anti-osteoporosis treatment and rehabilitation should also be standardized.

The optimal fixation method for patellar fractures remains debated, with plate osteosynthesis emerging as a viable alternative to traditional tension band wiring. According to Bickel et al, tension band wiring and locked plating showed comparable 1-year functional outcomes (21). Biomechanical analyses suggest that fixed-angle plate osteosynthesis of the patella offers significantly greater stiffness and lower fracture gap dehiscence than the other fixation methods (22). Siljander et al. reported favorable outcomes with low-profile mesh plates, demonstrating lower complication rates compared to tension band wiring, which is often associated with hardware irritation and secondary surgeries (23).

## 5 Conclusion

Compared to the other two groups, the ring-pins group demonstrated several advantages, including a broad range of applications, low postoperative complication rates, and ease of implant removal. This method provides a superior treatment experience for patients, characterized by low surgical complexity and favorable postoperative recovery of knee function, making the ring-pins technique a preferred option for patellar fracture surgery.

However, this study has certain limitations. As a retrospective analysis, surgical outcomes may have been influenced by variations in surgeons' skill levels. Additionally, the follow-up period was relatively short, requiring further evaluation of medium- to long-term outcomes. Future studies should incorporate extended follow-up durations to better evaluate the outcomes of these techniques.

## Data Availability

The raw data supporting the conclusions of this article will be made available by the authors, without undue reservation.
